# The Immediate and Long-Term Impact of Preeclampsia on Offspring Vascular and Cardiac Physiology in the Preterm Infant

**DOI:** 10.3389/fped.2021.625726

**Published:** 2021-05-31

**Authors:** Annabelle L. Frost, Katie Suriano, Christina Y. L. Aye, Paul Leeson, Adam J. Lewandowski

**Affiliations:** ^1^Oxford Cardiovascular Clinical Research Facility, Division of Cardiovascular Medicine, Radcliffe Department of Medicine, University of Oxford, Oxford, United Kingdom; ^2^Nuffield Department of Women's and Reproductive Health, University of Oxford, Oxford, United Kingdom

**Keywords:** preeclampsia, hypertensive pregnancy, gestational hypertension, preterm birth, cardiovascular risk, cardiac remodeling, vascular remodeling, endothelial function

## Abstract

Hypertensive disorders of pregnancy, including preeclampsia, affect nearly 10% of all pregnancies and are associated with significant long-term detrimental effects on both maternal and offspring cardiovascular health. Current management of preeclampsia involves timely delivery with the more severe form of disease requiring iatrogenic preterm birth. The effects on the maternal cardiovascular system have been studied extensively; however, less is known about the short- and long-term impacts on offspring cardiovascular health. There is a growing body of evidence suggesting that the offspring of pre-eclamptic pregnancies have an altered cardiac structure and function, along with a unique vascular physiology driven by lower endothelial function. Many of these changes can also be seen in those born preterm even in the absence of pregnancy hypertension. It is difficult to determine the relative contribution of pre-maturity and preeclampsia to the cardiovascular phenotype of those exposed to these pregnancy complications as they are, in many cases, inextricably linked. This review, therefore, focuses specifically on the evidence from clinical studies showing a negative cardiovascular impact of preeclampsia in preterm-born offspring, investigating phenotypic similarities and differences between offspring born preterm to normotensive vs. pre-eclamptic pregnancies. We explore the unique cardiac and vascular alterations in pre-eclamptic offspring born preterm, highlighting knowledge gaps, and potential areas of further research in the field.

## Introduction

Hypertensive disorders of pregnancy complicate up to 10% of pregnancies globally and account for at least 8–10% of all preterm births (1). They are defined by the new onset of hypertension after 20 weeks of gestation (2). Preeclampsia, which sits at the more severe end of the spectrum of hypertensive pregnancy disorders, includes in its definition signs of end organ dysfunction such as proteinuria, neurological symptoms, deranged biochemical markers of the hepatorenal and coagulation system, and/or of uteroplacental dysfunction (2). At present, the only definitive management of the condition is iatrogenic birth, which is preterm in some severe cases (1).

As well as an increased short-term morbidity and mortality in mother and child, it is now well-established that hypertensive disorders of pregnancy are associated with long-term sequelae. The mother is at an increased risk of early onset cardiovascular disease, with an ~50% increased risk of ischemic heart disease, five-fold increased risk of hypertension, three-fold increased risk of cardiovascular death, and approximately double the risk of heart failure and stroke during the decades after the pregnancy (3–6). In the offspring, preeclampsia has been shown to be an independent risk factor for hemorrhagic and thrombotic stroke, independent of preterm birth (7). Nevertheless, individuals who were born preterm have their own unique cardiovascular risk profile (8, 9), making it challenging to disentangle differences in cardiovascular characteristics between these populations. It is important to recognize that, although these populations overlap, differences in maternal pregnancy pathology may lead to different phenotypic characteristics in the offspring (10, 11). In this review, these differences are explored in order to provide a better understanding of the cardiovascular changes for each condition. This could allow for more targeted and appropriate clinical management and intervention strategies in the future.

## Underlying Vascular Alterations Differ Based on Maternal Pathology

Offspring of pre-eclamptic pregnancies have an increased risk of high blood pressure emerging in childhood (12) and double the risk of stroke in later life (13). This cardiovascular risk correlates with severity of disease, with early onset preeclampsia (occurring at <34 weeks gestation) being related to severe vascular dysfunction (14). Despite blood pressure elevation being a consistent finding in adolescents and young adults born to hypertensive pregnancies (15) and those born preterm (16), the underlying vascular alterations may differ depending on maternal pathology during pregnancy.

In a young adult study by Lazdam et al. (17), of *n* = 71 individuals born preterm (*n* = 52 to normotensive pregnancies and *n* = 19 to gestational hypertension or preeclampsia) and *n* = 38 term-born controls, underlying large vessel structural and functional differences between groups were explored. The study showed that brachial systolic blood pressure was, on average, >6 mmHg higher and brachial diastolic blood pressure >5 mmHg higher in preterm-born individuals born to hypertensive or normotensive pregnancies when compared with term-born controls. Differences were greater for central measures of blood pressure, with mean arterial pressure shown to be 8 mmHg higher in both preterm groups compared with those born at term. Regardless of maternal pregnancy pathology, preterm-born individuals had higher carotid intima media thickness compared with term-born individuals, though this was highest in those born preterm to hypertensive pregnancies. Interestingly, pulse wave velocity, a surrogate measure of arterial stiffness, was 6–9% higher in those born preterm to normotensive pregnancies compared with those born preterm to hypertensive pregnancies or born term, with no differences between the latter two groups. In contrast, brachial flow-mediated dilatation, a surrogate measure of large vessel endothelial function, was nearly 40% lower in young adults born preterm to hypertensive pregnancies compared with those born preterm to normotensive pregnancies or born term, with no differences between the latter two groups. These results suggest unique vascular abnormalities underlying blood pressure elevation depending on maternal pathology, with impairments primarily in endothelial function in those born preterm to hypertensive pregnancies.

Lawlor et al. (18) later published a study looking at brachial artery flow-mediated dilatation in children aged 9–12 years who were part of the Avon Longitudinal Study of Parents and Children. Of the *n* = 4,654 individuals who underwent vascular measures, *n* = 215 were born preterm, of which *n* = 25 were born to a pre-eclamptic pregnancy. The authors found no association between large artery endothelial function and hypertensive pregnancy status in either the individuals born preterm or those born at term. However, as the authors mentioned, the differences in age group could account for this negative finding, especially given that they were studied around the time of puberty. Puberty is a key developmental stage with significant hormonal changes and oxidative stress, known to have a particular impact on endothelial function and blood pressure during this period (19–21). Furthermore, timing of pubertal onset has been shown to vary in pre-eclamptic offspring (22). An additional possibility for variation between studies in children and young adults could relate to the higher representation of late preterm gestations in their preterm subgroup.

Another possibility for these potentially conflicting findings is that large artery endothelial functional differences in this population emerge later on in development as a downstream consequence of earlier microvascular remodeling and cellular endothelial alterations. Indeed, results from the Generation R study showed that higher maternal blood pressure levels during pregnancy associate with offspring retinal vascular changes in childhood (23). In a study of *n* = 60 children aged 7–12 years, of which *n* = 39 were born preterm, Bonamy et al. (24) showed that dermal capillary density was lower in the preterm group compared with term-born controls. Although the authors found that capillary density did not differ statistically significantly when comparing individuals born preterm to pre-eclamptic pregnancies vs. those born preterm to normotensive pregnancies, capillary density was, on average, 5% lower in the group of individuals born preterm to pre-eclamptic pregnancies. The lack of a statistically significant difference between groups could be due to small study numbers (only *n* = 17 of the *n* = 39 preterm-born individuals were born to pre-eclamptic pregnancies), with further confounding due to the presence of individuals in the preterm subgroup who suffered from retinopathy of pre-maturity. In a study by Lewandowski et al. (25) of young adults with a mean age of 25 years, it was also shown that preterm-born individuals had a lower dermal capillary density than term-born controls. Although *n* = 102 individuals born preterm and *n* = 102 born term were included in the study, only a subgroup underwent dermal capillary density measures (*n* = 30 born preterm and *n* = 60 born term). Given that <30% of the preterm cohort were born to pre-eclamptic pregnancies, differences based on maternal pathology could not be explored. However, circulating angiogenic and antiangiogenic markers, including soluble fms-like tyrosine kinase 1 (sFlt-1), were measured in all *n* = 204 individuals and shown to be higher in the preterm group compared with term-born controls, with a positive association between sFlt-1 in the offspring and maternal preeclampsia (25).

Studies in infancy have provided the majority of the evidence of altered angiogenesis and vasculogenesis in pre-eclamptic offspring. Muñoz-Hernandez et al. (26) studied *n* = 50 women, of which *n* = 15 had pre-eclamptic pregnancies, to determine whether endothelial colony-forming cells (ECFCs) from cord blood differed depending on pregnancy history. ECFCs are a late outgrowth form of endothelial progenitor cells with high vasculogenic potential (27), playing a key role in early vascular development. The authors demonstrated that cord blood ECFC levels were lower in pre-eclamptic pregnancies and took longer to emerge in culture. When looking specifically at individuals born preterm, normotensive ECFC levels were significantly higher than ECFC levels from preterm hypertensive pregnancy cord blood, and were also higher than ECFC levels from both term normotensive and hypertensive pregnancy cord blood. The elevation is consistent with previous reports on ECFC levels from preterm normotensive pregnancies (28). Pre-maturity status, however, did not affect ECFC levels in preeclampsia, as these levels were lower compared with normotensive controls in both the preterm and term groups. In addition, ECFC levels in cord blood were similarly reduced in both preterm hypertensive pregnancies and term hypertensive pregnancies.

Unique alterations in endothelial cells were further demonstrated in a study by Yu et al. (29) in a cohort of *n* = 255 infants (*n* = 104 born to normotensive pregnancies and *n* = 151 born to hypertensive pregnancies). It was shown that offspring of hypertensive pregnancies undergo greater postnatal microvascular remodeling. Normally, during early postnatal life, the disorderly fetal microvascular plexus remodels into a mature, horizontal papillary loop structure, leading to an overall decline in small vessel density (30). However, in this study, it was shown that the loss in total dermal microvascular density over the first 3 months of postnatal life was greatest in those born to hypertensive pregnancies, especially in those born -term. However, this microvascular density loss was similar in offspring of preterm hypertensive pregnancies and preterm normotensive pregnancies. A subgroup of *n* = 55 individuals, of which *n* = 24 were born to hypertensive pregnancies, also had umbilical cords collected with human umbilical vein endothelial cells (HUVECs) extracted. Reductions in *in vitro* vascular tube formation and branching points were seen in HUVECs from both term and preterm hypertensive pregnancies, and in multivariable regression models, hypertensive pregnancy was the only independent predictor of these changes. Interestingly, sFlt-1 levels circulating in venous blood from the women, which were higher in women from hypertensive pregnancies, correlated positively with both *in vivo* and *in vitro* microvascular reductions in the offspring.

In a follow-on study, Yu et al. (31) investigated possible mechanisms, focusing on microRNAs given their important role as endothelial cell regulators. MicroRNA-146a (miR-146a) was identified as a top candidate from unbiased RNA sequencing and confirmed by quantitate reverse transcription polymerase chain reaction. Multivariable regression analysis was undertaken and demonstrated that the elevation in miR-146a was independent of other perinatal factors including gestational age and was unique to hypertensive pregnancies. The elevation in miR-146a was associated with reduced *in intro* and *in vivo* microvasculature, which could be rescued in HUVECs by miR-146a inhibition. Importantly, overexpression of miR-146a in HUVECs from normotensive pregnancies caused a reduction in vascular tube formation. Taken together, these findings show that, although similar microvascular reductions may be present in preterm-born individuals irrespective of maternal pathology, the underlying mechanisms may differ, with primarily endothelial alterations in those born preterm to hypertensive pregnancies.

## Cardiac Remodeling: a Unique Contribution of Preeclampsia on the Preterm Heart

Preeclampsia, especially in the context of preterm delivery, is commonly characterized by impaired placental perfusion and a high-resistance maternal uterine circulation compared with normotensive pregnancies (32). The alterations in resistance and flow could lead to a disruption of cardiac cell signaling, growth, and chamber remodeling during early development (33). In spite of this, few studies have investigated the impact of preeclampsia and gestational hypertension on offspring cardiac structure and function.

In a study by Aye et al. (34) that included *n* = 255 individuals with repeat neonatal and infant echocardiography scans, individuals born preterm (*n* = 121, of which *n* = 70 were born to hypertensive pregnancies) were found to have unique postnatal cardiac remodeling patterns compared with term-born controls. Preterm birth is associated with a disproportionate increase in left ventricular (LV) and right ventricular (RV) mass during the first months of postnatal life. At 3 months of infancy, preterm offspring had double the percentage of postnatal mass change for the LV when compared with term-born infants. Preterm neonates also showed a two-fold increase in RV mass index. Similarly, LV diastolic function decreased relative to term-born controls during the first three postnatal months, with persistent reductions in RV systolic function during this period. While the majority of these structural and functional impairments could be predicted by the degree of pre-maturity, hypertensive pregnancy was an independent predictor of the increase in RV mass during the first three postnatal months. In order to investigate the effect of pregnancy hypertension alone on cardiovascular structure, Aye et al. (35) performed a follow-on study in *n* = 134 term-born individuals, of which *n* = 80 were born to either preeclampsia or gestational hypertension. The study demonstrated similar LV and RV masses in both groups at birth; however, term-born infants born to hypertensive pregnancies had persistently smaller RV end-diastolic volumes (16.8 ± 5.3 vs. 12.7 ± 4.7 ml/m^2^, *P* = 0.001), which persisted into infancy (16.4 ± 3.2 vs. 14.4 ± 4.8 ml/m^2^, *P* = 0.04). At 3 months of postnatal life, infants born to hypertensive pregnancies also showed subtle changes in LV mass (24.9 ± 4.6 vs. 26.8 ± 4.9 g/m^2^, *P* = 0.04) and greater changes in RV mass at 3 months of postnatal life (17.1 ± 4.2 vs. 21.1 ± 3.9 g/m^2^ *P* < 0.001). These findings were similar to those by Timpka et al. (36) in a follow-up study of *n* = 1,592 adolescents aged 17 years in the Avon Longitudinal Study of Parents and Children. Although primarily term-born individuals, those born to pre-eclamptic pregnancies (*n* = 42) and gestational hypertension (*n* = 247) had greater LV relative wall thickness, with smaller LV end-diastolic volumes in the pre-eclamptic group.

Although the preterm heart has been characterized in a recent meta-analysis of 32 individual studies showing unique alterations in cardiac remodeling from birth through to adulthood (37), including smaller LV end-diastolic volumes, lower LV diastolic and RV systolic function across developmental stages, as well as an accelerated rate of LV hypertrophy from childhood to young adulthood, the majority of studies did not differentiate between preterm normotensive and preterm hypertensive pregnancies. In a study by Lewandowski et al. (38) that included *n* = 102 preterm-born young adults aged 25 years, it was shown that LV longitudinal peak systolic strain, a measure of LV systolic function reflecting changes in loading conditions that is affected by myocardial contractility (39), was impaired compared with term-born young adult controls. When looking within the preterm group, those born preterm to pre-eclamptic pregnancies (*n* = 29) had a further 9% reduction in LV longitudinal peak systolic strain compared with individuals born preterm to normotensive pregnancies (*n* = 73). Given that impairments in strain, and specifically longitudinal strain, often pre-date global systolic functional impairments, such as ejection fraction, and have important predictive and prognostic implications in cardiovascular disease (40), further research is needed to specifically explore this. Many of these cardiovascular alterations are seen in offspring of animals with pre-eclamptic-like pregnancies (13, 41), which may offer the opportunity to further explore therapeutic targets and interventions. Whether deficits in LV function seen in preterm-born young adults born to normotensive pregnancies during the physiological stress of exercise (42, 43) are further impaired in those born preterm to pre-eclamptic pregnancies remains to be determined but is plausible given the further reductions in systolic function seen at rest.

## Discussion

**Figure 1:**
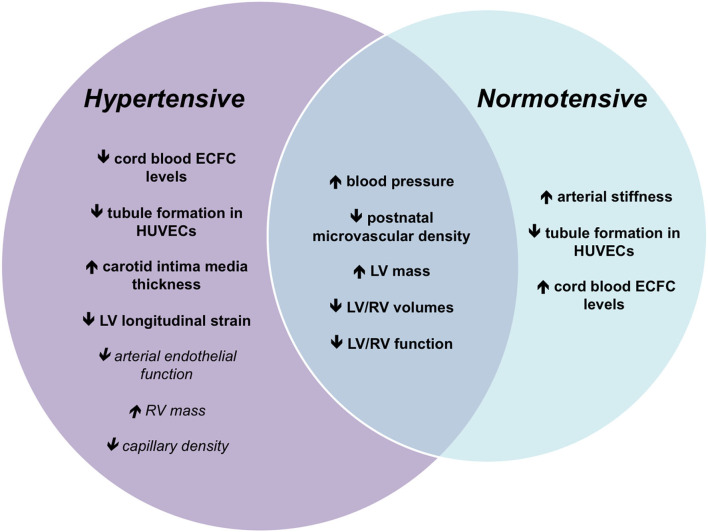
Venn diagram showing the unique cardiac and vascular changes in offspring born preterm to normotensive and hypertensive pregnancies. All characteristics described differ from those born at term to normotensive pregnancies. Changes shown in the middle of the Venn diagram are not distinguishable between the offspring born preterm to normotensive and hypertensive pregnancies. Changes on the left are additional changes seen in the offspring born preterm to hypertensive pregnancies, and those on the right are additional changes seen in the offspring born preterm to normotensive pregnancies. Characteristics on the left that are italicized and not in bold are inconclusive and require further investigation. ECFC, endothelial colony-forming cell; HUVEC, human umbilical vein endothelial cell; LV, left ventricle; RV, right ventricle.

At present, the number of studies reporting on the vascular and cardiac structure and function in offspring of pre-eclamptic pregnancies born preterm with an appropriate comparison group of individuals born preterm to normotensive pregnancies remains limited. Nevertheless, it appears that vascular impairments in preterm-born individuals born to preeclampsia and gestational hypertension have a unique underlying physiology primarily characterized by endothelial dysfunction. Furthermore, some adverse changes in LV and RV structural and functional changes are also distinctive in offspring born to preeclampsia and gestational hypertension, which are also present even in the absence of pre-maturity (Figure 1).

A significant challenge to the field arises due to collider stratification bias, where associations with later outcome may be driven by causes of preterm birth rather than pre-maturity *per se* (44). Further research studies with adequate sample size to specifically study long-term cardiovascular changes related to preeclampsia and gestational hypertension are needed. Ideally, these studies should include repeated measures on the same individuals throughout development to better understand the life course of vascular and cardiac alterations in this population. These steps will be essential for developing appropriate lifelong screening and cardiovascular prevention models.

## Author Contributions

AL and PL conceptualized the original review topic. AF and AL drafted the initial article, reviewed and revised the article, and approved the final article as submitted. KS, CA, and PL critically reviewed and revised the articles and approved the final version for submission. All authors contributed to the article and approved the submitted version.

## Conflict of Interest

The authors declare that the research was conducted in the absence of any commercial or financial relationships that could be construed as a potential conflict of interest.
